# Three Cases of Sympathetic Irido-Cyclitis

**Published:** 1884-04

**Authors:** L. B. V. Woolley


### THREE CASES OF SYMPATHETIC IRIDO-CYCLITIS.

FROM THE CLINIC OF A. W. CALHOUN, M. D.,

Professor of Diseases of Eye, Ear, and Throat, Atlanta Medical College.

REPORT BY L. B. V. WOOLLEY, M. D.

Case I. Wm. B——, aged 18 years, admitted October 12, 1883. From an injury to the right eye, irido-cyclitis set up resulting in the total destruction of the function of that eye. The vision in the left eye had been gradually failing. There was evidently irido-cyclitis in the left eye, the result of sympathy. As the right eye was destroyed nothing of course was hoped to be accomplished except the arrest of the disease in the left eye. This was accomplished by the removal of the source of irritation, the injured eye.

The patient being etherized, a circular incision was made with a pair of blunt pointed, curved scissors, in the conjunctival tissue near the corneo-sclerotic juncture. After opening with the scissors the subconjunctival tissue over the insertions of the various muscles of the ball, they were seized with forceps and severed near their insertions. The curved scissors being closed were passed backward from the temporal side until the optic nerve was reached, when they were opened and the nerve and accompanying artery were seized and severed near the sclerotic. The order in which the muscles and nerve were cut was the external rectus, superior and inferior recti muscles, the optic nerve, the superior and inferior oblique and internal rectus muscles. The hemorrhage, which was not profuse, was soon stopped by sponges dipped in cold water, and slight compression. The dressing consisted in a piece of soft linen about an inch square, greased and placed in the bottom of the wound, at the apex of the orbital cavity. Over this was placed a pledget of cotton to fill the vacancy left by the removed ball, the lid being pulled down over this and a handkerchief tied over the eye. The patient was directed to bathe the eye frequently with cold water for a day or two. This dressing was removed in forty-eight hours and not renewed.

The lids were kept separated during the operation with an eye speculum. The conjunctiva, at the temporal side, was seized in the beginning with a pair of fixation forceps. This hold should not be released until the ball is removed. In the dressing the greased cloth is of utmost importance, for without it the lint will adhere to the wound and be exceedingly painful and difficult to remove. The stump of muscular conjunctival and other tissue that is left in the operation affords an excellent support for an artificial eye, while it also renders it capable of much muscular control.

This patient was dismissed in one week after the operation, the wound having healed and sight of the remaining eye much improved. He was directed to drop in the left eye, three times a day, three or four drops of an astringent collyrium (borac pulv. gr. iv. *et* morph. sulph. gr. ij *ad* aq. camph. ℥i.)

Case II. Mrs. S. A. L.———, aged 65 years, admitted October 25, 1883. About ten months previous there was ulceration of the right cornea. Seven or eight months afterwards hypopyon appearing, paracentesis corneæ was resorted to. The sight in this eye was totally destroyed. There was evidence of irido-cyclitis in the left eye, the result of sympathy. The right eye was enucleated as in the last case described. The patient made a good recovery in eight or ten days.

Case III. H. L———, aged 50 years, admitted December 20, 1883. About two years previous, from an explosion of a gun, the right eye was injured. The ball had collapsed. In about two months after the injury the sight in the left eye began to fail. This failure had continued until he could count fingers removed only three feet. There was evidence of irido-cyclitis in the left eye, the result of sympathy. The same operation made as above described. The operation proved a success and the patient was dismissed in eight or ten days.

There is no good to be had by temporizing in these cases. Nothing will suffice, when the disease has once set up, but the removal of the offending object by enucleation, or other operative interference.

